# Preferential crystallization for the purification of similar hydrophobic polyphenols

**DOI:** 10.1002/jctb.5526

**Published:** 2018-01-31

**Authors:** Marcelo Silva, Briana Vieira, Marcel Ottens

**Affiliations:** ^1^ Delft University of Technology Department of Biotechnology Delft The Netherlands

**Keywords:** crystallization, process optimization, downstream, purification, mathematical modeling

## Abstract

**BACKGROUND:**

Preferential crystallization is a common technique used in the purification of enantiomers, proving that crystallization may also be applied to the purification of very similar molecules by seeding the solution with the desired compound. Nonetheless, its application to other organic molecules is less widely documented in the literature. Knowing that chemically related polyphenols are generally co‐produced by fermentation and their purification can be too expensive for their market value, this technique may contribute to developing a downstream process with less expensive steps. The goal of this work is to show the applicability of the preferential crystallization concept to the purification of similar polyphenols – naringenin and trans‐resveratrol – with either single or coupled crystallizers.

**RESULTS:**

After developing the required crystallization kinetic models, an experiment using two coupled vessels was devised, where a 63% yield of naringenin and 44% yield of trans‐resveratrol was obtained, with ≥98% purity in both cases. When the vessels were working independently, 81% of pure trans‐resveratrol (started 60% pure) and 70% of pure naringenin (started 68% pure) were recovered.

**CONCLUSION:**

The experiments performed show the possibility of separately purifying two similar molecules (from 60% to roughly 100%) with promising yields, despite their similar solubility. This method, which can be significantly improved, might provide an economically attractive way for the production of low added value products. © 2017 The Authors. *Journal of Chemical Technology & Biotechnology* published by John Wiley & Sons Ltd on behalf of Society of Chemical Industry.

## NOMENCLATURE

**Table:** 

Variable	Name	Units
B_2, i_	Secondary nucleation rate of polyphenol i	# cm^‐3^ min^‐1^
$k_{N2,i}^{0}$	Secondary nucleation parameter	# cm^‐3^ min^‐1^
E_N2, i_	Activation energy for secondary nucleation	J mol^‐1^
R	Universal gas constant	J K^‐1^ mol^‐1^
b	Secondary nucleation parameter	‐
M_T_	Total mass of crystals in suspension	kg L^‐1^
j	Secondary nucleation parameter	‐
m	Momentum order of the crystal size distribution	‐
L	Characteristic crystal length	μm
Δc	Supersaturation level	kg kg^‐1^ suspension
Δc_us_	Undersaturation level	kg kg^‐1^ suspension
G_i_	Growth rate of polyphenol i	μm min^‐1^
$k_{G}^{0}$	Crystal Growth parameter	μm min^‐1^
E_G_	Activation energy for crystal growth	J mol^‐1^
p	Crystal Growth parameter	‐
D_i_	Dissolution rate of polyphenol i	μm min^‐1^
$k_{D}^{0}$	Crystal Dissolution parameter	μm min^‐1^
E_D_	Activation energy for crystal dissolution	J mol^‐1^
q	Crystal Dissolution parameter	‐
${\hat{\mu }}_{m,i}$	m ^th^ distribution moment of polyphenol i	# μm^m^ L^‐1^
${\bar{n}}_{i}$	Relative number of crystals of polyphenol i in suspension	μm^‐1^
N_i_	Total number of crystals of polyphenol i in suspension	#
ρ_c, i_	Crystal density	kg m^‐3^
k_v, i_	Crystal volume shape factor	‐
${\bar{k}}_{v,i}$	Average crystal volume shape factor	‐
k_v, i0_	Parameter for describing k_v, i_(L)	μm^‐x^
x	Exponent for describing k_v, i_(L)	‐
t_seed_	Time point at which seed crystals are added to the vessel	h
$C_{l,i}^{k}$	Liquid concentration of polyphenol i in vessel k	g L^‐1^
$C_{s,i}^{k}$	Solid concentration of polyphenol i in vessel k	g L^‐1^
$C_{l,i,\mathrm{nk}}^{\mathrm{tub}}$	Liquid concentration of polyphenol i inside tubing coming from vessel n to vessel k	g L^‐1^
$C_{0,l,i}^{k}$	Initial liquid concentration of polyphenol i in vessel k	g L^‐1^
$C_{0,s,i}^{k}$	Initial solid concentration of polyphenol i in vessel k	g L^‐1^
$C_{f,l,i}^{k}$	Final liquid concentration of polyphenol i in vessel k	g L^‐1^
$C_{f,s,i}^{k}$	Final solid concentration of polyphenol i in vessel k	g L^‐1^
m_seed_	Mass of seed crystals of a given polyphenol	g
$m_{0,i}^{k}$	Initial mass of polyphenol i in vessel k	g
F_nk_	Flow rate from crystallizer n to crystallizer k	L h^‐1^
V^k^	Liquid volume in vessel k	L
T	Liquid temperature	°C
T_t_	Actual temperature in the thermostat, after applying a given set‐point	°C

## INTRODUCTION

Polyphenols are molecules which have a range of different biotechnological applications (e.g. as food additives, nutraceuticals, and food colorants).^1^ Most of these molecules are secondary metabolites produced naturally by plants, and they are composed of multiple phenol structural units. Over recent years, research on their health properties has grown considerably,^2^ with authors studying the properties of these molecules in the prevention of diseases such as Alzheimer's and several types of cancer.^3^ Furthermore, the increasing interest in these compounds has led to the creation of projects such as the BacHBerry project,^4^ funded by the 7th Framework Programme of the European Commission. This project aimed to discover new phenolic compounds with interesting properties (e.g. health‐promoting, colorants) and develop a sustainable process for their production using bacterial platforms. The downstream process development for the capture and purification of polyphenols produced in such a way is then crucial for the success of the project.

The production of these molecules, either by fermentation or by extraction from diverse plant material, is likely to result in the release of similar polyphenols in solution. These structurally close molecules might pose a considerable challenge for the downstream process design since more selective operations should be needed. Since food additives tend to have a low market price and polyphenol fermentation processes normally have low yields,^5^ the design and optimization of alternative purification strategies is of paramount importance.

**Figure 1:**
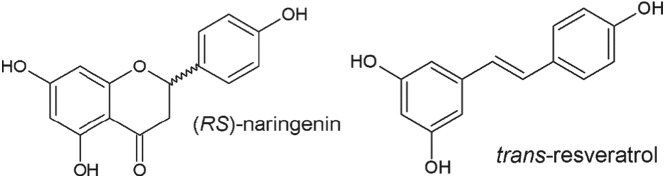
Chemical structure of naringenin and trans‐resveratrol.

Preferential crystallization is a purification technique first used in 1853 by Pasteur^6^ for the resolution of sodium ammonium tartrate tetrahydrate enantiomers, and since then, it has been used for similar purposes.^6^, ^7^, ^8^ The basic underlying idea of this technique is that crystallization can achieve purification not only because of a difference between the thermodynamic driving forces (i.e. different solubility curves) but also due to differences in the compounds' crystallization kinetics. By seeding a crystallizer containing a liquid solution with the desired compound, conditions are applied which promote the growth of crystals of the desired molecule and its consumption from the liquid phase, while the impurity only starts crystallizing when its supersaturation level is sufficiently high to start nucleation.^6^ Despite its logical application to conglomerate forming enantiomers, the applicability of preferential crystallization can be extended to other organic molecules.^9^ In this work, preferential crystallization using two coupled vessels is investigated as a method to purify structurally similar, hydrophobic polyphenols, with overlapping solubility curves – naringenin and trans‐resveratrol (Fig. 1) – after a hypothetical preliminary purification step using reverse‐phase adsorption. It will be assumed that trans‐resveratrol leaves the adsorption step with a purity of 60% in a 39% w/w ethanol solution and that naringenin is collected in a fraction with 60% purity as well, but in 46% w/w ethanol. This different ethanol content aims to mimic what would happen in a typical gradient elution profile in chromatography. In order to optimize the applied crystallization controls (temperature in each vessel and flow rates), the kinetics of crystallization was modeled by describing secondary nucleation, crystal growth, and crystal dissolution. For estimating the required kinetic parameters, different batch experiments were performed, where different quantities of seed crystals were used, and different temperature profiles were applied, which also included varying the heating and cooling rates. The models developed were then used to optimize the preferential crystallization conditions in the coupled vessel experiment.

## MATERIAL AND METHODS

### Theoretical background

The major goal of this work was to set‐up a coupled vessel preferential crystallization experiment for the purification of naringenin and trans‐resveratrol. In order to optimize the yields obtained, the temperature in each vessel was continuously controlled as well as the flow rates pumped from each crystallizer to the other. By applying filters in both vessels, only the liquid phase was assumed to be pumped from one vessel to another. In order to determine those optimal controls, the approach taken in this work was to model the crystallization kinetics by performing batch experiments. The models obtained were then transferred to the coupled vessel situation, where the predicted yields of both polyphenols were maximized while constraining their purity to be at least 95%.

In this section, the crystallization kinetic models used for both batch and coupled vessel experiments are introduced. The component mass balances are also described for each case.

#### Crystallization kinetics

For modeling crystallization kinetics, phenomena like nucleation, crystal growth, and crystal dissolution have to be described mathematically. The overall goal was to obtain a model that would describe the experimental observations as well as possible, but preserve simplicity. For that reason, primary nucleation was disregarded, since experiments were run at relatively low supersaturation levels and seed crystals were always present. Moreover, crystal aggregation was not considered as well, because the expected improvement in modeling prediction would not compensate for the added complexity.

For modeling secondary nucleation, an empirical power law was used, considering that the most important variables involved in this process are supersaturation, temperature, solid content and rotational speed of the propeller:^10^

(1) $$B_{2,i}=k_{N2,i}^{0}e^{\left(-\frac{E_{N2,i}}{\mathrm{RT}}\right)}{\left({\Delta c}^{b}\right)}_{i}{\left(M_{T}^{j}\right)}_{i}$$

Since the agitation speed was kept constant throughout all the experiments, its impact on the secondary nucleation rate was not included explicitly, but included in the kinetic constant $k_{N2,i}^{0}$.

Concerning the description of crystal growth, it was assumed that no dependence on the crystal characteristic size, L, occurred. Thus, a structurally similar empirical expression to the one provided for secondary nucleation was used:^10^, ^11^

(2) $$G_{i}=k_{G,i}^{0}e^{\left(-\frac{E_{G,i}}{\mathrm{RT}}\right)}{\left({\Delta c}^{p}\right)}_{i}$$

This equation incorporates, in the p exponent, the combined effect of diffusion and surface integration into the growth kinetics.^10^

By analogy with the growth rate kinetics, the rate of dissolution was described by the same model, but with the undersaturation level replacing supersaturation:^11^

(3) $$D_{i}=k_{D,i}^{0}e^{\left(-\frac{E_{D,i}}{\mathrm{RT}}\right)}{\left({\Delta c}_{\mathrm{us}}^{q}\right)}_{i}$$

#### Mass balances for the liquid and solid phases

In order to describe the concentration of each polyphenol in both liquid and solid phases, the method of moments was applied.^12^, ^13^ Taking into account that the liquid volume might vary with time – in the case of the coupled vessel experiments – the equations for the 0th and higher moments are given by:^12^

(4) $$\frac{d\left({\hat{\mu }}_{0,i}\cdot V^{k}\right)}{\mathrm{dt}}=B_{2,i}\cdot V^{k}$$

(5) $$\frac{d\left({\hat{\mu }}_{m,i}\cdot V^{k}\right)}{\mathrm{dt}}=mG_{i}\left({\hat{\mu }}_{m-1,i}\cdot V^{k}\right)$$

In these equations, k stands for the vessel number (k = 1, 2), as for the coupled vessel experiment the liquid volume in each vessel may be different.

At time zero, seed crystals with known particle size distribution are added to the crystallizer. Because the particle size distribution determined was obtained using the relative frequency (${\bar{n}}_{i}$), it is necessary to multiply by the total number of crystals in suspension, in order to obtain the mth moment of the absolute particle distribution (volume specific):

(6) $${\hat{\mu }}_{m,i}\left(t=0\right)=\frac{N_{i}}{V^{k}}\int_{0}^{\infty }\left(L_{i}^{m}\cdot {\bar{n}}_{i}\right)\;\mathrm{dL}$$

Once the crystal size distribution is known at time zero and the differential equations for the evolution of its moments over time are known (Equations (4) and (5)), the mass balance for the liquid phase can be described by Equations (7) and (8). Their use depends on whether the solution is supersaturated or undersaturated:

(7) $$\frac{d\left(C_{l,i}\cdot V^{k}\right)}{\mathrm{dt}}=-3\cdot \rho_{c,i}\cdot {\bar{k}}_{v,i}\cdot G_{i}\cdot \left({\hat{\mu }}_{2,i}\cdot V^{k}\right)$$

(8) $$\frac{d\left(C_{l,i}\cdot V^{k}\right)}{\mathrm{dt}}=3\cdot \rho_{c,i}\cdot {\bar{k}}_{v,i}\cdot D_{i}\cdot \left({\hat{\mu }}_{2,i}\cdot V^{k}\right)$$

These equations basically state that an eventual increase or decrease of dissolved compound mass is consumed in either the crystal growth or dissolution process. In both of them, the average volume shape factor of the crystals, ${\bar{k}}_{v,i}$, is introduced. The inconvenience with this formulation is that the volume shape factor will depend on the crystal characteristic length (shown in the following section). Since the method of moments was preferred due to its simplicity, avoiding solving partial differential equations, a consistent way of defining the average volume shape factor was devised: it was taken as that of the seed crystals used in each experiment. The underlying assumption was that the number of crystals in suspension were in sufficient number that crystal growth would have a negligible impact on crystal size (a small growth in a large crystal population consumes as much supersaturation as a large growth in a small population). How this average was calculated and used to take into account the fact that the aspect ratio of the crystals depends on their characteristic size L, is shown in the next section.

Similarly to how the previous mass balances were written, the mass balance of the solid phase is symmetric:

(9) $$\frac{d\left(C_{s,i}\cdot V^{k}\right)}{\mathrm{dt}}=3\cdot \rho_{c,i}\cdot {\bar{k}}_{v,i}\cdot G_{i}\cdot \left({\hat{\mu }}_{2,i}\cdot V^{k}\right)$$

(10) $$\frac{d\left(C_{s,i}\cdot V^{k}\right)}{\mathrm{dt}}=-3\cdot \rho_{c,i}\cdot {\bar{k}}_{v,i}\cdot D_{i}\cdot \left({\hat{\mu }}_{2,i}\cdot V^{k}\right)$$

When both crystallizers are connected, an inlet filter of 10 μm is put on each tubing inlet, and an inline filter of 0.2 μm is put on the discharge side of each peristaltic pump so that it is assumed that only liquid phase is exchanged between them. Due to the liquid flow between each vessel, the liquid volume is expected to vary according to the following differential equation:

(11) $$\frac{dV^{k}}{\mathrm{dt}}=F_{\mathrm{nk}}-F_{\mathrm{kn}}$$

In this equation, F_nk_ stands for the flow rate coming from vessel n to vessel k.

Because the tubing used had a significant dead volume (approximately 9 mL, compared with a 100 mL liquid volume inside each crystallizer), it could affect the crystallization model predictions, if not taken into account. Thus, the flow inside the tubing interconnecting both crystallizers was modeled as a plug flow:

(12) $$\frac{\partial \left(C_{l,i,\mathrm{nk}}^{\mathrm{tub}}\right)}{\partial t}=-\frac{F_{\mathrm{nk}}}{A_{\mathrm{tub}}}\;\frac{\partial \left(C_{l,i,\mathrm{nk}}^{\mathrm{tub}}\right)}{\partial x}$$

In this equation, x is the coordinate axis pointing in the direction of the liquid flow. The tubing internal area, A_tub_, was calculated from the tubing internal diameter of 3.1 mm provided by the supplier (data from Masterflex).

The fact that liquid can be pumped across the two different reactors has two implications. First, when the experiments are run, both crystallizers start with different ethanol in water concentrations. Because of that, as liquid is transported from one vessel to the other, that ethanol concentration changes with time. Second, as it was assumed that no solid material can pass through the filters, the major difference between Equation (13) and Equation (7) is that the mass of dissolved product not only can change due to crystal growth/dissolution but also by being transported from one vessel to another. Thus, for the liquid phase:

(13) $$\frac{\partial \left(C_{l,i}^{k}.V^{k}\right)}{\partial t}=F_{\mathrm{nk}}\;C_{l,i,\mathrm{nk}}^{\mathrm{tub}}-F_{\mathrm{kn}}\;C_{l,i}^{k}-3\cdot \rho_{c,i}\cdot {\bar{k}}_{v,i}\cdot G_{i}\cdot \left({\hat{\mu }}_{2,i}\cdot V^{k}\right)$$

For the solid phase, because no solid material is assumed to be transferred, the balance remains unchanged:

(14) $$\frac{\partial \left(C_{s,i}^{k}.V^{k}\right)}{\partial t}=3\cdot \rho_{c,i}\cdot {\bar{k}}_{v,i}\cdot G_{i}\cdot \left({\hat{\mu }}_{2,i}\cdot V^{k}\right)$$

As indicated in all the equations presented, describing the kinetics of crystallization requires knowing both supersaturation and undersaturation levels at each time point. Because both temperature and ethanol concentration might vary with time, knowledge of the solubility curves of both polyphenols as a function of those two variables is essential. Using experimental data already available in the literature,^13^, ^14^ the Jouyban–Acree model^15^ was used in order to have an explicit solubility function:

(15) $$\begin{array}{cc} T\cdot \ln\left(x_{i}\right) & =A_{0}+A_{1}T+A_{2}Tx_{\text{EtOH}}+A_{3}x_{\text{EtOH}}+A_{4}x_{\text{EtOH}}^{2}\\ & +A_{5}x_{\text{EtOH}}^{3}+A_{6}x_{\text{EtOH}}^{4} \end{array}$$

In this equation, x_i_ is the molar fraction of polyphenol i, x_EtOH_ is the ethanol molar fraction in solution and A_0_ to A_6_ are regressed parameters.

#### Temperature control

When solving the optimal control problem, it is considered that the user defines a temperature set‐point at defined time intervals. However, because the thermostat does not reach the desired set‐point instantaneously, but rather takes some time to reach it, two types of equations were used to describe the time taken by the thermostat to warm or cool the liquid solution to the desired level.

For heating, a linear model was sufficient to describe the observations:

(16) $$T_{t}=a+b\cdot t$$

For cooling, a quadratic model provided a better fit to the data obtained (data not shown):

(17) $$T_{t}=c+d\cdot t+e\cdot t^{2}$$

In both equations shown, T_t_ represents the actual temperature of the thermostatic bath. The parameters a, b, c, d and e were estimated by performing heating and cooling rate experiments with the thermostats used. In each of the experiments performed, it was determined how much time each thermostat would take to reach a certain set‐point after it was defined.

### Chemicals

Milli‐Q ultrapure water was available through an ultrapure water system provided by Merck Millipore. The ethanol used was EMSURE Ethanol absolute for analysis. The polyphenol trans‐resveratrol was provided by Evolva SA, a part of Olon S.P.A. (Italy), with a purity ≥98%. Naringenin, natural (US) with a purity of 98% was provided by Sigma‐Aldrich.

### Seed crystal preparation

To prepare seed crystals of trans‐resveratrol, the polyphenol was first dissolved in a solution of 39% (w/w) ethanol/water. Once dissolved, the solution was filtered and deposited in a wide Petri dish. The filtered solution was then transferred to the oven at 60 °C. After approximately 2.5 h, the solution containing crystals was taken out of the oven and stored in a flask for further use, letting the temperature equilibrate with room temperature. To prepare seed crystals of naringenin, the compound was first dissolved in a solution of 46% (w/w) ethanol/water at 60 °C and put in a crystallization vessel. The temperature was then decreased to –5 °C at a rate of –10 °C h^‐1^. After crystal formation, the suspension was filtered and the crystals resuspended in an aqueous solution saturated with naringenin. At the end of the process, the slurry was stored in a flask for further use. The only exception was for the coupled vessel experiment, where instead of filtering, the slurry was re‐heated to 20 °C to achieve room temperature and stored afterward.

The crystals of naringenin were prepared by cooling, instead of evaporation, in order to prevent agglomeration as much as possible. However, for the crystal density determination, the crystals were prepared as was mentioned for trans‐resveratrol.

In both cases above‐mentioned, the solid content was measured by mass balance most of the time (knowing the initial and final volumes and liquid phase concentrations). For the coupled preferential crystallization experiment, the solid content was measured by vacuum filtering a known volume of seed crystal solution and determining the solid weight.

### Particle size distribution measurement

The particle size distributions were measured using a Leica DM5500B microscope (Leica Microsystems GmbH, Germany) combined with an automated image analysis software (LEICA Qwin). In order to analyze particle sizes offline, samples were taken using a pipette with a cut tip to prevent clogging and in order to have a representative size distribution of the sample. The samples were then observed under the microscope, and several pictures were taken. The developed script using the LEICA Qwin software removed the background noise and counted only the single crystals. Since agglomerates were observed to be composed of multiple individual needle crystals together (Figure in Supplementary material), it was considered that their size distribution was the same as the one determined when considering only single crystals.

### Crystal density measurement

The crystal density was measured by using a 50 mL volumetric flask and then measuring the volume displaced by a given mass of crystals. This method, based on volume displacement, is described elsewhere.^16^ The solution used for filling the flask was a saturated aqueous solution of the respective polyphenol, at room temperature, in order to avoid any dissolution. The obtained densities were 1.37 ± 0.07 g mL^‐1^ for naringenin and 1.4 ± 0.1 g mL^‐1^ for trans‐resveratrol.

### Experimental set‐up

For the estimation of the crystallization kinetic parameters, batch experiments were performed in a single vessel with double wall glass (product code 6.1418.250) from Metrohm Applikon (Netherlands). The liquid temperature was controlled using a cooling thermostat (RE 307 Ecoline star edition from LAUDA).

The agitation, fixed at 250 rpm, was provided by a Hei‐TORQUE 100 overhead stirrer from Heidolph Instruments GmbH & Co. KG (Germany) and a mini‐propeller from Bohlender GmbH (Germany).

**Figure 2:**
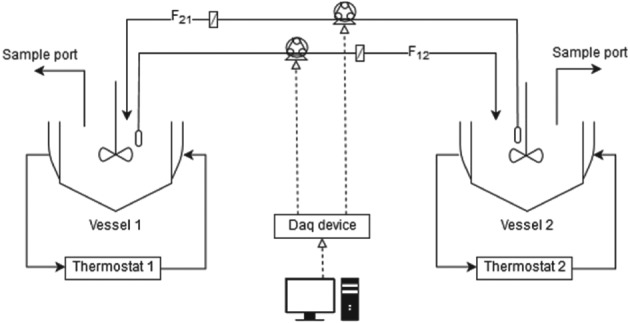
Scheme of the coupled crystallizer set‐up for the preferential crystallization experiments.

For the preferential crystallization experiment with two coupled vessels, there were two similar double wall batch crystallizers as described above. Moreover, there were 10 μm inlet filters and 0.2 μm inline filters at the pump discharge side, to avoid pumping crystals that might have gone through the tubing. The flow rate was controlled by a NI USB‐6001 DAQ device from National Instruments, connected to two Masterflex® pumps, model 77521‐57. The tubing used was Chem‐Durance Bio Pump Tubing, L/S 16, from Masterflex®. The experimental set‐up is depicted in Fig. 2.

### Sampling and polyphenol quantification

Samples were taken with cellulose acetate 0.2 μm filter in order to prevent crystal suction and then measured by UHPLC (Ultimate 3000, Thermo Scientific, USA) in a C18 column (Acquity UPLC HSS column, 1.8 μm, 2.1 mm x 100 mm Waters, Milford, USA). Mobile phase A (10% formic acid in Milli‐Q water) and mobile phase B (10% formic acid in acetonitrile) were run through the column at a constant flowrate of 0.4 mL min^‐1^. Every run was performed in isocratic mode, containing 33.5% of mobile phase B and 66.5% of mobile phase A. The detection of trans‐resveratrol was performed at 304 nm and that of naringenin at 289 nm.

## RESULTS AND DISCUSSION

### Solution density

Since the temperature during the experiments varied from –5 °C to 50 °C, the density of both ethanol and water had to be modeled as a function of temperature. For that, the DIPPR105 equation in the DDBST GmbH database was used:^17^

(18) $$\rho =\frac{A}{B^{1+{\left(1-\frac{T}{C}\right)}^{D}}}$$

The A, B, C and D parameters in this equation do not have any relation to the crystallization parameters previously defined. They correspond to the parameters provided in the DDBST GmbH database, for describing the densities of both water and ethanol (Table 1). The density of water/ethanol mixtures was considered to be the density of the pure components, weighted by their mass fraction.

**Table 1:** Parameters for the DIPPR105 equation, describing how the solvent density changes with temperature

Solvent	A	B	C	D
Ethanol	99.374	0.310729	513.18	0.305143
Water	0.14395	0.0112	649.727	0.05107

### Solubility data fitting

The solubility data of both naringenin and *trans*‐resveratrol as a function of ethanol concentration and temperature is available from the literature.^13^, ^14^ This data was then used in order to regress the parameters needed for the Jouyban–Acree model, as described earlier. This equation describes the solubility as a function of temperature and the co‐solvent molar fraction (ethanol in this case). For the case of naringenin, the experimental data between 0.1 ethanol molar fraction up to 0.4 was used (the range of interest). For *trans*‐resveratrol, the solubility data up to 0.4 molar fraction was considered (also within the range of interest). The parameters obtained for the Jouyban‐Acree model were obtained by minimizing the sum of squared relative errors, as it was observed that the uncertainty of the experimental data increased with the solubility absolute value. The regressed parameters are indicated in Table 2.

**Table 2:** Jouyban–Acree parameters for modeling the solubility of trans‐resveratrol and naringenin as a function of the ethanol content and temperature

Parameter	trans‐resveratrol	naringenin
A0	(–6.0 ± 0.4) × 10^3^	(–6.0 ± 0.2) × 10^3^
A1	7 ± 1	6.2 ± 0.7
A2	–15 ± 4	–6 ± 3
A3	(2.0 ± 0.2) × 10^4^	(1.42 ± 0.08) × 10^4^
A4	(–3.7 ± 0.6) × 10^4^	(–2.73 ± 0.02) × 10^4^
A5	(4.3 ± 0.5) × 10^3^	(3.68 ± 0.01) × 10^3^
A6	(–1.8 ± 0.7) × 10^3^	(–2.289 ± 0.006) × 10^3^
RMSE	2.3 × 10^‐4^	9.3 × 10^‐5^

**Figure 3:**
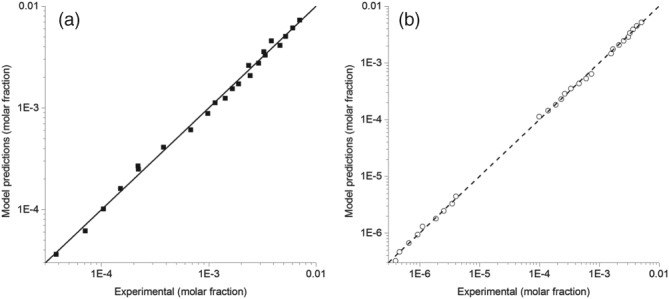
Comparison between the experimental solubility data and the Jouyban–Acree model predictions. The results for trans‐resveratrol are shown in figure 3a and those of naringenin in figure 3b.

The comparison between experimental data and model predictions are indicated in Fig. 3. As can be observed, the predictions match quite well the literature data, as is also implied by the low RMSE values of the model.

### Single vessel preferential crystallization and determination of maximum supersaturation levels

**Figure 4:**
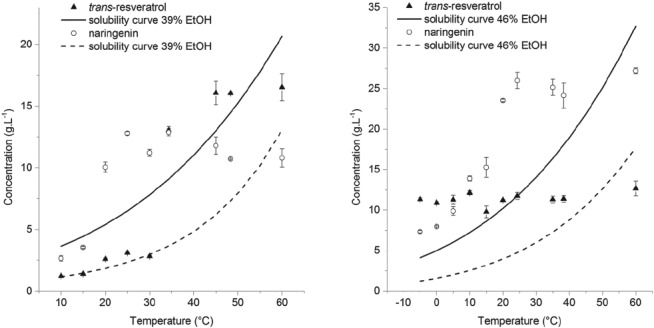
Progress of the preferential crystallization experiment performed in a single vessel. On the left is shown the experiment performed with a 39% w/w solution of ethanol, where trans‐resveratrol starts with a purity of 60%. On the right, an analogous experiment was performed, starting with naringenin 60% pure in a solution of 46% w/w ethanol. The error bars indicate the standard deviation in the UHPLC concentration measurements**.**

For the preferential crystallization to be successful, a maximum supersaturation level has to be defined in order to prevent crystallization of the unwanted polyphenol. For setting those limits in the coupled vessel experiment, preferential crystallization experiments in a single vessel were performed, where either *trans*‐resveratrol or naringenin was initially present with 60% purity (the 40% impurity consisted of the other polyphenol). After dissolving both compounds and allowing the temperature to decrease, in order to achieve some supersaturation, seed crystals of the desired polyphenol were added. The temperature was then allowed to decrease at a rate of –10 °C h^‐1^ and the liquid concentration of both polyphenols monitored over time. The results are shown in Fig. 4.

For the case of *trans*‐resveratrol, one can observe that naringenin (in this case, the impurity) only starts nucleating and growing at approximately 20 °C, where the supersaturation ratio (liquid concentration divided by solubility) is around 4.5. For the analogous experiment with naringenin, *trans*‐resveratrol appeared not to have reached sufficient supersaturation to start nucleating. Nonetheless, a maximum level of supersaturation ratio was set at 2.6.

At this point, it is also worth mentioning that these experiments provided a proof‐of‐concept for the use of preferential crystallization. Although there was still no coupling between both vessels, it was observed that even above the solubility curve of the undesired polyphenol, nucleation only occurred considerably after the crystals of the desired molecule started growing. For the case represented in Fig. 4 (on the left), *trans*‐resveratrol started with 61% purity and, when the temperature reached 25 °C, the solid phase attained 81% yield at a purity of around 100% (no naringenin crystallizing). In Fig. 4 (on the right), naringenin started 68% pure and, when at 0 °C, the solid phase had reached 70% yield of naringenin with approximately 100% purity (still no *trans*‐resveratrol crystallizing).

### Crystal volume shape factor determination

In order to account for the non‐cubic shape of the crystals, their volumetric shape factor had to be calculated. In both cases, it was observed that both crystals were needle‐like shaped (although naringenin crystals tended to aggregate). For that reason, their shape was approximated as a rectangular parallelepiped.

It was observed under the microscope that the preferential growth direction of the crystals was along the parallelepiped length (*L*). Thus, that length *L* was chosen as the characteristic length of the crystal. Because growth not only occurred in length but also in width (*W*), the shape factor, *k*_*v*_, was expected to vary with *L*. For the rectangular parallelepiped approximation:

(19) $$k_{v,i}\left(L\right)={\left(\frac{W}{L}\right)}^{2}$$

In order to model the dependence of the shape factor on the characteristic length, a generic power model seemed to properly describe the experimental data:

(20) $$k_{v,i}\left(L\right)=k_{v,i0}\cdot L^{x}$$

The regressed parameters (*k*_*v*,*i*0_ and *x*) for both polyphenols are indicated in Table 3.

**Table 3:** Regressed parameters for the Equation (19), which relates the crystal volumetric shape factor as a function of the crystal characteristic length

Polyphenol	*k*_*v,*0_	*v*
naringenin	1.4 ± 0.1	–1.62 ± 0.02
*trans*‐resveratrol	23 ± 2	–2.09 ± 0.02

**Figure 5:**
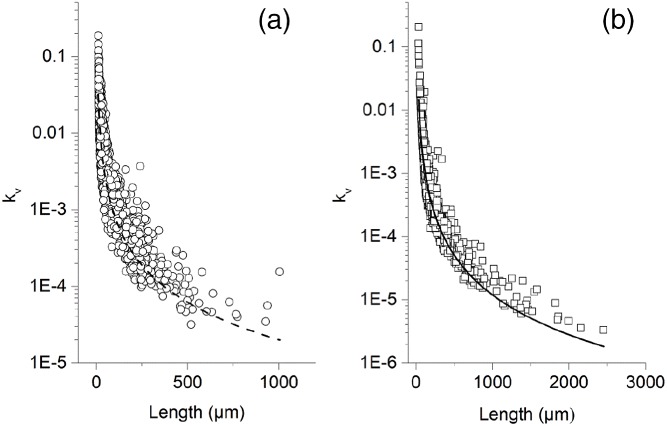
Volumetric shape factor of trans‐resveratrol (a) and naringenin (b) as a function of their characteristic length.

The model fit to the experimental data is depicted in Fig. 5:

To use the volume shape factor in the method of moments, the value should not have a dependence on the characteristic length. As previously explained, due to the fact that the calculation of ${\bar{k}}_{v}$ could not be coupled with the characteristic length (it would be necessary to solve partial differential equations), an approximation had to be made. In this work, the average crystal shape factor was calculated from the particle size distribution of the seed crystals:

(21) $${\bar{k}}_{v,i}=\int_{L_{0}}^{L_{f}}\bar{n}\cdot k_{v,i}\;\mathrm{dL}$$

### Crystallization kinetic parameters estimation

For modeling the time evolution of polyphenol concentration in the crystallizer, the kinetic parameters associated with secondary nucleation, growth and dissolution still had to be determined. In order to do so, four experiments in a single batch crystallizer were devised for each polyphenol. For each of them, different temperature profiles were applied, and different mass of seed crystals was used (values are indicated in each graph). The range of temperature covered was the range of interest for the preferential crystallization experiment using coupled vessels. Thus, for both *trans*‐resveratrol and naringenin, the temperature was varied between –5 °C and 50 °C.

**Figure 6:**
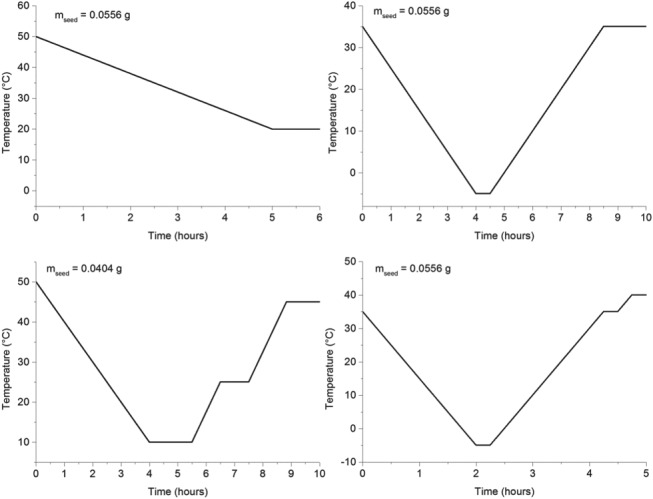
Temperature profiles used for the single batch experiments using trans‐resveratrol. Also indicated in each graphic is the mass of seed crystals introduced so that crystal growth was promoted**.**

For the case of *trans*‐resveratrol, the following temperature profiles were applied (see Fig. 6).

The goal of these experiments was to cool and heat the solution at different rates, in order to achieve different supersaturation and undersaturation levels over time. The reasoning behind this is that regarding optimal experimental design, the higher those levels, the more ‘information’ is obtained for determination of the kinetic parameters. That can be derived from the mathematical description of secondary nucleation, growth, and dissolution, which consists of a power function of either supersaturation or undersaturation.

**Figure 7:**
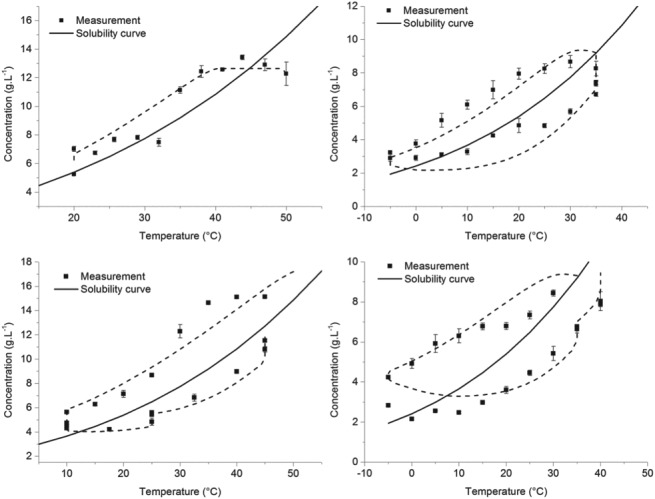
Progress of the liquid phase concentration of trans‐resveratrol, during each of the four batch crystallization experiments previously indicated. Model predictions are shown as dashed lines and the solubility curve in 46% w/w ethanol solution is shown as a full line. The error bars indicate the standard deviation in the UHPLC concentration measurements**.**

After regressing the 10 kinetic parameters associated with the batch crystallization experiments performed (Table 4), the comparison between the liquid concentration profiles obtained experimentally and those provided by the model are shown in Fig. 7.

**Table 4:** Regressed kinetic parameters for trans‐resveratrol

Parameter	Regressed value
$k_{N2}^{0}$	2.09 × 10^8^ *
*E*_*N*2_	5.00 × 10^6^ *
*b*	4.05*
*j*	3.05*
$k_{G}^{0}$	(6.6 ± 0.3) × 10^6^
*E*_*G*_	(3.00 ± 0.01) × 10^4^
*p*	(5.819 ± 0.002) × 10^‐1^
$k_{D}^{0}$	(5.84 ± 0.01) × 10^8^
*E*_*D*_	(3.5113 ± 0.0009) × 10^4^
*q*	1.1521 ± 0.0002

* **The standard deviation is too large, as secondary nucleation seemed to not be important for the applied conditions.**

As can be observed, the kinetic models used seem to be able to follow quite well the liquid concentration profile of the polyphenol. Nonetheless, the confidence intervals on the secondary nucleation parameters of *trans*‐resveratrol were larger than their average value (Table 4). This result suggests that, during the experiments performed, secondary nucleation might not have had a significant impact on the supersaturation levels applied. To confirm that was the case, the same model was run, but assuming no secondary nucleation would take place (all the remaining parameters were kept unchanged). The model obtained was indistinguishable from the first one (data not shown). A possible explanation for this has to do with the supersaturation level attained in the reactor. Unlike most of the cooling crystallization processes at industrial scale, where most compounds have solubility between 100 and 300 g L^‐1^, 18 the solubility of the polyphenols used in this work is at most around 15 g L^‐1^. Because of that, provided the cooling rate is low enough, the supersaturation in the system should be consumed mostly for crystal growth and not for secondary nucleation (higher supersaturation levels are usually needed for significant secondary nucleation to occur).

**Figure 8:**
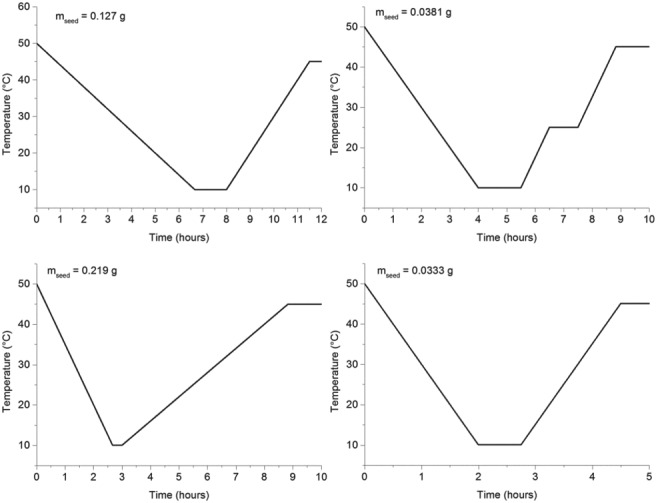
Temperature profiles used for the single batch experiments using naringenin. Also indicated in each graphic is the mass of seed crystals introduced so that crystal growth was promoted**.**

For the case of naringenin, the applied temperature profiles are shown in Fig. 8. The goal when designing these experiments was the same as in the case before. However, because naringenin required higher supersaturation levels to start growing, the applied cooling profiles were quite similar in all the experiments. In order to circumvent that limitation and to try to achieve different supersaturation/undersaturation levels during each experiment, different masses of seed crystals were added at the beginning of each experiment.

**Figure 9:**
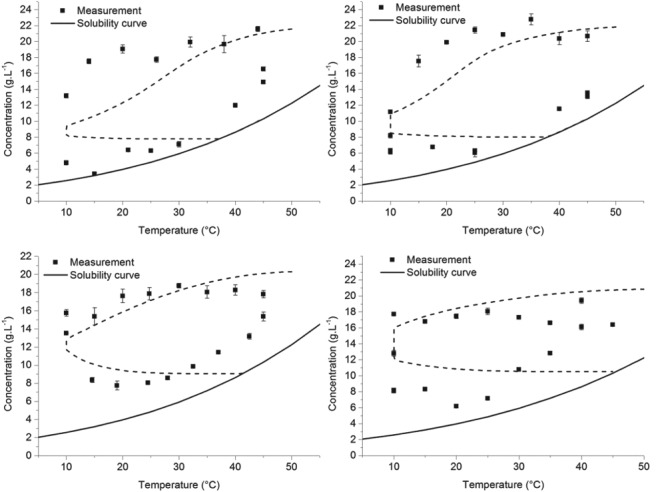
Progress of the liquid phase concentration of naringenin, during each of the four batch crystallization experiments previously indicated. Model predictions are shown as dashed lines and the solubility curve in 46% w/w ethanol solution is shown as a full line. The error bars indicate the standard deviation in the UHPLC concentration measurements.

The same comparison between experimental values and model predictions like the one performed for *trans*‐resveratrol is indicated in Fig. 9.

When comparing with trans‐resveratrol, significant differences could be detected in the behavior of naringenin. First, it was observed that agglomeration was occurring with time, which was not accounted for in the more simple kinetic model used. On the other hand, the level of supersaturation needed for growth to occur was significantly higher than the one for trans‐resveratrol, despite seed crystals being added at the beginning of the experiment. By looking at Table 5, which contains the regressed parameters for naringenin, it is possible to check that the growth exponent, p, is 4.19, much higher than the value of 0.58 for trans‐resveratrol. This difference might be for several reasons. First, there is some uncertainty associated with the solubility curve determination, which was interpolated using the Jouyban–Acree model. Second, a polynuclear growth mechanism might be present, which is associated with a larger growth rate exponent.^19^ The last explanation proposed is related to the occurrence of agglomeration itself, observed in the experiments performed and that was not accounted for. Since the models used had the goal of being able to predict the liquid concentration profile over time while keeping simplicity, they may not be able, as in this case of naringenin, to fully describe the physical and chemical aspects of the crystallization process. Because of that, the regressed parameters in Table 5 had too small a confidence region, but to which no statistical meaning was given.

**Table 5:** Regressed kinetic parameters for naringenin. The confidence intervals are not provided, since they do not provide a reasonalbe statistical interpretation of their degree of uncertainty

Parameter	Regressed value
$k_{N2}^{0}$	9.33 × 10^30^
E_N2_	3.87 × 10^3^
b	5.76
j	3.13
$k_{G}^{0}$	2.59 × 10^9^
E_G_	2.39 × 10^3^
p	4.31
$k_{D}^{0}$	1.75 × 10^11^
E_D_	1.70 × 10^4^
q	1.36

**Figure 10:**
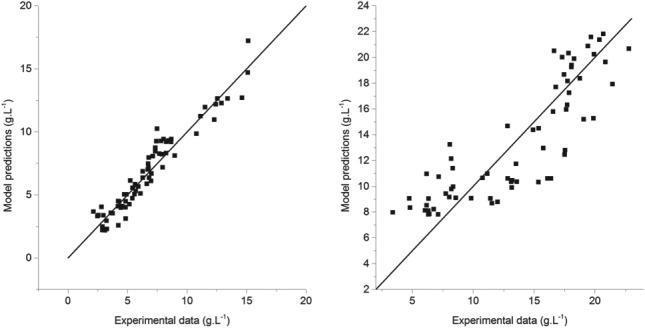
Comparison between the experimental liquid concentration data and the crystallization kinetic model for trans‐resveratrol (left) and naringenin (right).

Figure 10 shows the comparison between model predictions and experimental data: it is possible to determine that the systems containing only trans‐resveratrol are better described than the ones containing naringenin, possibly for the reasons detailed before.

### Preferential crystallization experiment using coupled vessels

As was previously shown, the concept of preferential crystallization was validated by performing two independent batch experiments, where both polyphenols were initially present, but only the seeded compound was able to grow to a certain level of supersaturation of the impurity. In order to improve the yield of both compounds in their respective vessel, a strategy using two coupled vessels was executed.^8^ In this experiment lasting for 6 h, both the temperatures in each vessel as well as the flow rates were controlled every 20 mins. In order to approach the optimal process conditions, an optimal control problem was formulated:

(22) $$\underset{T_{1}\left(t\right),\;T_{2}\left(t\right),F_{12}\left(t\right),\;F_{21}\left(t\right)}{\;\text{max min}}\left(Y_{\mathrm{naringenin}},Y_{\mathrm{trans}-\mathrm{resveratrol}}\right)$$

In this expression, *Y*_*naringenin*_ and *Y*_*trans* − *resveratrol*_ correspond to the yields of the respective polyphenol. In each reactor, the temperature controls could not be set below 0 °C or above 50 °C.

(23) $$0\leq T\left({}^{{}^{\circ}}C\right)\leq 50$$

The flow rates being delivered by the pumps were also subject to constraints. The minimum was set as the minimum flow rate the pump could deliver (1 mL min^‐1^). The maximum was set at 5 mL min^‐1^, to ensure that a small offset between the set‐point and the flow rate actually delivered would not cause overfilling of the vessels:

(24) $$1\leq F_{\mathrm{ij}}\left(\mathrm{mL}\;{\text{min}}^{-1}\right)\leq 5$$

Additional constraints were set as indicated in Equation (25). The liquid volume in each vessel should not be lower than 60 mL or higher than 150 mL, to avoid overfilling the vessel or having too low a volume to be stirred. The boundaries imposed on the supersaturation ratios were obtained from the previous preferential crystallization experiments using single vessels (2.6 for *trans*‐resveratrol and 4.5 for naringenin). The liquid temperature was also required to be always equal to or larger than the temperature at the time when the seed crystals are added, in order to avoid their dissolution. The solid content was also limited to a maximum of 20 g L^‐1^ because with too high a concentration, the inlet filter started to become covered with solid mass causing the pump to cavitate, not delivering the desired flow.

(25) $$\left[\begin{array}{c} T\left(t\geq t_{\text{seed}}\right)\leq T\left(t_{\text{seed}}\right)\\ 60\leq V\left(\mathrm{mL}\right)\leq 150\\ {\mathrm{SSR}}_{\text{resv},1}\leq 2.6\\ {\mathrm{SSR}}_{\mathrm{nar},2}\leq 4.5\\ C_{s}\left(g\cdot L^{-1}\right)\leq 20 \end{array}\;\right]$$

In Equation (25), *SSR*_*resv*, 1_is the supersaturation ratio of *trans*‐resveratrol in vessel 1 and *SSR*_*nar*, 2_ the supersaturation ratio in vessel 2. The formulated optimal control problem was solved using Matlab and the NOMAD algorithm.^20^, ^21^, ^22^, ^23^

**Figure 11:**
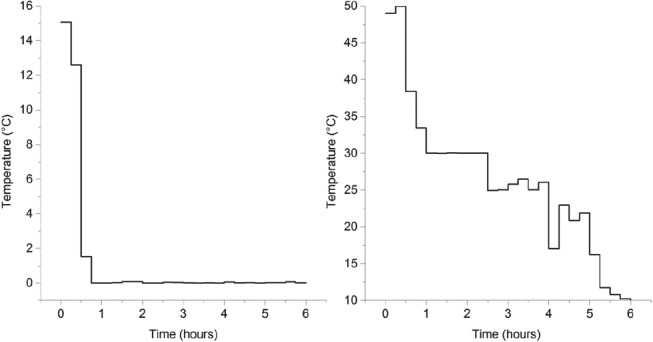
Optimal temperature set‐points for crystallizer 1, where naringenin is preferentially crystallized (left) and crystallizer 2 (right), where trans‐resveratrol is preferentially crystallized. The unexpected jumps in the temperature level might be due to the existence of several local minima**.**

The optimal temperature controls obtained after performing the optimization are indicated in Fig. 11.

Owing to the high nonlinearity of this problem, it is expected that multiple minima can exist. For the temperature control in crystallizer 1 (Fig. 11, on the left), the solution obtained starts, almost from the beginning, asking for a temperature close to 0 °C. This is because naringenin requires a high supersaturation level to start growing and also because, at that temperature, *trans*‐resveratrol is still below a supersaturation ratio of 2.6 (the set constraint in order to avoid primary nucleation). On the other hand, in vessel 2, the temperature starts by slowly decreasing until 25 °C. At this point, the model assumes that the crystals of *trans*‐resveratrol can grow while keeping naringenin below a supersaturation ratio of 4.8. After approximately 3.5 h, when the crystallizers are exchanging fluid because the concentration of naringenin is supposed to lower (more dilute naringenin solution comes from vessel 1, and more concentrated solution leaves vessel 2), the temperature is further reduced to 10 °C. The goal is to increase yield and keep high purity.

**Figure 12:**
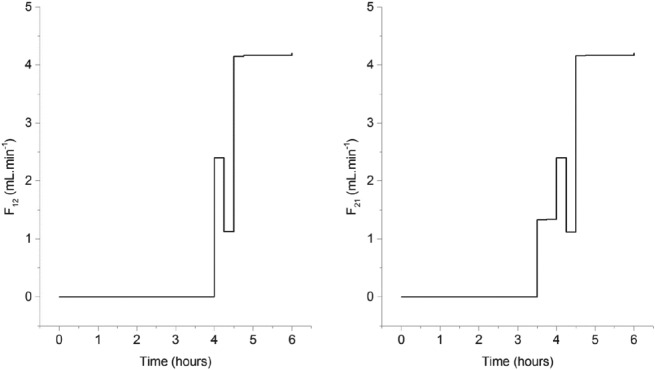
Optimal flowrate set‐points for crystallizer 1, where naringenin is preferentially crystallized (left) and crystallizer 2 (right), where trans‐resveratrol is preferentially crystallized**.**

The optimum controls obtained for the flow rates exchanged by each vessel are indicated in Fig. 12.

As can be observed, the pumps were only started after approximately 3.5 h of experiment. This is because it only makes sense to start pumping when the solution becomes richer in the impurity. Vessel 2 ends up with a volume of 60 mL and vessel 1 with 140 mL. This is because the model assumes that the most difficult polyphenol to crystallize, given the constraints, is naringenin (the supersaturation ratio of *trans*‐resveratrol has to be below 2.6). Because the minimum yield is being maximized and increasing the yield of *trans*‐resveratrol is ‘easier’, the mathematical solution obtained asks for more volume in vessel 1 in order to crystallize more naringenin (the limiting polyphenol). Based on the determined controls, the Matlab script developed predicted a 78% yield of *trans*‐resveratrol and a 68% yield of naringenin, both reaching a final purity of 100%.

**Figure 13:**
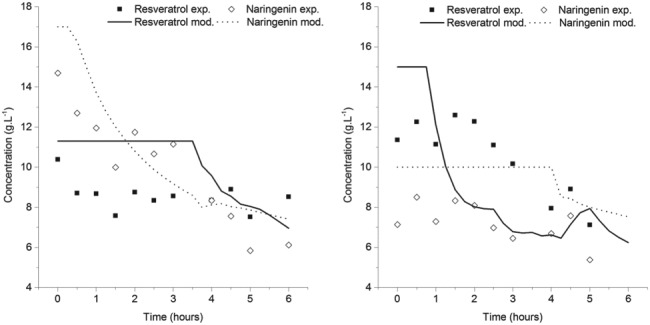
Liquid concentration of both naringenin and trans‐resveratrol over time, for crystallizer 1 (left) and crystallizer 2 (right). The model predictions are indicated in full and dashed lines in order to provide a comparison with the experimental data obtained.

The experiment was performed using the coupled crystallizer scheme, and the results are plotted in Fig. 13.

Two analyses are made in this section of the results depicted in Fig. 13. First, regarding the vertical offset between the model predictions and the experimentally obtained values, the difference can be explained by the impact of the cellulose acetate filter on the sampling process. For instance, the initial concentration of naringenin in vessel 1 was determined to be 17.7 g L^‐1^ without using the filter and 14.7 g L^‐1^ when using the filter. The same happened with *trans*‐resveratrol and in both solutions. Although the sampling process may have a certain impact on the absolute concentration determination, the same protocol was followed in all experiments and thus, all the model predictions should be consistent. Moreover, the error in the concentration determination should be considerably lower than the model uncertainty, which does not take into account aspects like agglomeration or the effect of the impurities on the growth rates. Considering now how the model predicts the liquid concentration progress over time for both polyphenols, it can be observed that naringenin in vessel 1 is quite well described, while resveratrol seems to remain essentially constant, although the model predicts that some dilution should occur after 3.5 h (fluid is coming from vessel 2). Since almost no *trans*‐resveratrol crystallized in vessel 1 (naringenin purity is close to 100%), the suggested explanation is that the *trans*‐resveratrol coming from vessel 2 is more concentrated than predicted by the model, as is possible to check in Fig. 13, on the right. In vessel 2, the description of *trans*‐resveratrol seems to have a horizontal offset, before there is any fluid exchange between the vessels (after 3.5 h). The proposed explanation is that naringenin might be acting as a growth inhibitor impurity, thus increasing the supersaturation needed for *trans*‐resveratrol to start growing. The concentration profile of naringenin is qualitatively well predicted, the only difference being more towards the end, where it seems to be more diluted than expected. The reason for that, since no naringenin crystallized in vessel 2 (see Table 6), is that naringenin being transported from vessel 1 is also more dilute than predicted by the model.

**Table 6:** Yield and purity of each polyphenol in both vessels 1 and 2, at the end of the preferential crystallization experiment.

Vessel	Yield*	Yield**†**	Predicted yield	Purity
1 (naringenin)	24%	63%	64%	98%
2 (*trans*‐resveratrol)	6%	44%	78%	100%

* Yield obtained using filtration.

† Yield obtained using mass balance.

The final yield of each compound was calculated using Equation (26), where the final solid mass was obtained by vacuum filtration.

(26) $${\text{Yield}}_{i}^{k}=\frac{\text{final mass of polyphenol in vessel}\;i-m_{\text{seed}}}{C_{0,l,i}^{k}V_{0}}$$

The term ${\text{Yield}}_{i}^{k}$ denotes the yield of polyphenol *i* in the vessel *k*.

Alternatively, the yield was calculated by mass balance. For the generic case of a polyphenol *i* in vessel 1, the expression used is indicated in Equation (27):

(27) $${\text{Yield}}_{i}^{1}=\frac{\left(C_{0,l,i}^{1}+C_{0,l,i}^{2}\right)V_{0}-\left(C_{f,l,i}^{1}V_{f,1}+C_{f,l,i}^{1}V_{f,2}\right)-C_{f,s,i}^{2}V_{f,2}-m_{\text{seed}}}{m_{0,i}^{1}}$$

The purity of the solids in both vessels was determined by UHPLC and using Equation (28). In this equation, the desired polyphenol corresponds to compound *i* and the impurity is represented by *j*.

(28) $${\text{Purity}}_{i}^{k}=\frac{C_{f,s,i}^{k}}{C_{f,s,i}^{k}+C_{f,s,j}^{k}}$$

The results obtained are compiled in Table 6.

As can be observed, there is a significant discrepancy between the yields calculated by mass balance and when measuring the solids after filtering the slurry.

First, the difference between the yield obtained by mass balance and the one predicted by the model is assumed to be mainly due to the uncertainty associated with the regressed parameters and to the fact that those parameters were estimated from single component crystallization experiments. Regarding the difference between the yield calculated by mass balance or by weighing the total final solid mass, the reason might be associated with crystal loss inside the tubing and on the inline filter. Due to their shape, despite the fact that needle crystals may be larger than 10 μm in their characteristic length, their width and depth might be low enough, so that they can enter the inlet filter through different angles. Moreover, knowing that liquid velocity inside the tubing is relatively low (in the range of 2×10^‐3^ m s^‐1^) and especially as the crystals grow, they might become more difficult to transport with the pumped fluid. Because they probably get blocked at the inline filter (0.2 μm pore size), they may tend to either accumulate at the filter or attach to the tubing walls (tubing has a dead volume of around 9 mL). Since this mass of crystals was not taken into account, it might explain the difference between the two different ways of calculating the yield. One aspect that supports this reasoning is that the liquid concentration of *trans*‐resveratrol in vessel 2 effectively decreased, as shown in Fig. 13 (figure on the right). However, by the time both vessels were exchanging liquid at the same rate, the concentration of this molecule on both vessels was similar, so no dilution effect should be present.

In the end, the goal of the coupled vessel experiment was to prove that it was possible to perform an offline process optimization using previously developed crystallization kinetics models and, within a certain error margin, to predict the time evolution of the liquid concentration of both polyphenols. Another aim was to suggest that, given some process changes (e.g. different filter pore sizes, preventing filter clogging, using online process control), this strategy should give a yield at least as large as the one obtained by using two uncoupled vessels. One possible way of overcoming these hurdles observed at the lab scale would be to use a continuous filtration unit between the two vessels. This would not only avoid passing solid material to the other vessel but also keep the solids concentration within the desired level.

## CONCLUSIONS

In this work, the applicability of preferential crystallization towards the recovery and purification of two similar polyphenols, naringenin and *trans*‐resveratrol, was tested. First, it was assumed that a preliminary purification, based on reverse‐phase adsorption, had been executed after a fermentation step. This purification step would have provided two fractions, each with a 60% purity of both naringenin and *trans*‐resveratrol. The preferential crystallization would then be a possible further purification unit, where the purity of each polyphenol would increase to at least 95%, and the minimum yield would be maximized.

The strategy followed in this work had three steps: first, performing the necessary batch experiments with the pure compounds in order to estimate their crystallization kinetic parameters (secondary nucleation, growth and dissolution); second, the optimal control problem was formulated in Matlab and solved in order to maximize the minimum polyphenol yield, while obeying certain constraints such as purity; last, the preferential crystallization experiment using two coupled vessels was executed and the results compared with the model predictions. One of the main conclusions obtained from this work is that preferential crystallization proved to be a feasible method of purifying two similar compounds, up until the point that the supersaturation level of the impurity leads to its nucleation. This was observed with the batch experiments performed, where either naringenin or *trans*‐resveratrol started with a 60% purity level. The goal here was to evaluate the maximum supersaturation level that would make the undesired polyphenol start nucleating. In fact, these experiments were no different from a coupled vessel preferential experiment with no fluid being exchanged between the two vessels. The second conclusion is related to the crystallization kinetic models developed. It was shown that for the case of *trans*‐resveratrol the model can predict quite well the liquid phase concentrations. Nonetheless, for naringenin, the model predictions were not as good, probably because that the underlying assumptions, such as no aggregation or no crystal growth dependence on the characteristic size, were not correct. However, the model obtained still had the capability of providing reasonably good predictions of the naringenin concentration in the liquid phase and, in the end, to prove its applicability to solve the final optimal control problem. The last point is related to the actual coupled preferential crystallization experiment. The results obtained showed the relative success of this set‐up, by increasing the purity of both polyphenols to approximately 100%, despite the relatively low yields (44% for *trans*‐resveratrol and 63% for naringenin, when calculated by mass balance). Although some discrepancy between the model predictions and the experimental concentration values was observed, the models developed had relative success in predicting the qualitative behavior of the crystallization progress. However, in order to explore the full potential of this technique, using a continuous filtration unit in between the two crystallizers could be more efficient. In that way, not only crystals could not pass to the other crystallizer, but also the solids concentration would be kept at the desired level in each vessel. In the end, the major goal of this study was to reinforce the idea that preferential crystallization can be applied for cases other than the resolution of racemic compounds and that this method can also be an economical way of purifying polyphenols, if they are closely related to each other, making it a better alternative to a possibly more expensive chromatography step.

## Supporting information

Figure S1.
