# Supplementary figures and images for: Preferential crystallization for the purification of similar hydrophobic polyphenols
Source: J Chem Technol Biotechnol. 2018 Jan 31;93(7):1997–2010. doi: 10.1002/jctb.5526 (PMC6033112; doi:10.1002/jctb.5526)

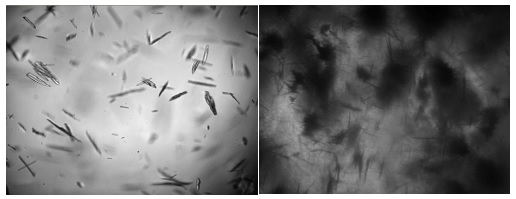

Supplement: Supplementary file 1 — Figure S1. [file JCTB-93-1997-s001.jpg]
